# Er,Cr:YSGG Laser-Activation Enhances Antimicrobial and Antibiofilm Action of Low Concentrations of Sodium Hypochlorite in Root Canals

**DOI:** 10.3390/antibiotics8040232

**Published:** 2019-11-22

**Authors:** Pablo Betancourt, Josep María Sierra, Octavi Camps-Font, Josep Arnabat-Domínguez, Miguel Viñas

**Affiliations:** 1 Laboratory Molecular Microbiology & Antimicrobials, Department of Pathology & Experimental Therapeutics, Faculty of Medicine, University of Barcelona, 08907 Barcelona, Spain; pablo.betancourt@ufrontera.cl (P.B.); jmsierra@ub.edu (J.M.S.); 2 Research Centre for Dental Sciences (CICO), Department of Integral Adult Dentistry, Universidad de La Frontera, Temuco 4810296, Chile; 3 Department of Dentistry, Faculty of Medicine, University of Barcelona, 08193 Barcelona, Spain; ocamps@ub.edu (O.C.-F.); joseparnabat@ub.edu (J.A.-D.)

**Keywords:** root canal infection, *Enterococcus faecalis*, biofilm, Er,Cr:YSGG laser

## Abstract

The onset and persistence of endodontic infections due to residual biofilm after chemical disinfection promotes secondary bacterial infection. Alternative methods to disinfect operated root canals are a matter of great interest. The aim was to evaluate the antibacterial effectiveness of sodium hypochlorite (NaOCl) at low concentrations activated by the Er,Cr:YSGG laser-activated irrigation (LAI) against 10-day-old intracanal *Enterococcus* *faecalis* biofilm. Biofilms were formed inside the root canals and divided into 7 groups (n13): 0.5% NaOCl + Er,Cr:YSGG; Saline + Er,Cr:YSGG; 0.5% NaOCl + syringe irrigation(SI); 2.5% NaOCl + SI; 5% NaOCl + SI; positive and negative controls. Bacterial survivors were counted and specimens visualized under scanning electron and confocal laser scanning microscopy. Treatments with 0.5% NaOCl + Er,Cr:YSGG and 2.5% NaOCl + SI gave a significant reduction in the number of CFU/mm^2^. Moreover, scanning electron microscopy and confocal laser scanning microscopy imaging confirmed and reinforced bacteriological data. Thus, Er,Cr:YSGG LAI proved to be able to improve the intracanal distribution of 0.5% NaOCl after 60 s of activation, reaching the same level of effectiveness than 2.5% NaOCl. This is regarded as of clinical interest, since working with lower concentrations may contribute to reduce undesired effects.

## 1. Introduction

The onset and persistence of endodontic infections due to residual biofilm after chemical disinfection promotes secondary bacterial infection [1]. Environmental conditions provided in the root canal favor polymicrobial growth. Nevertheless, *Enterococcus faecalis* is a frequently recovered bacterium from persistent infections [2]. *E. faecalis* is an aerotolerant anaerobic Gram-positive coccus, expressing several virulence factors, such as aggregation substances, enterococcal surface protein (Esp), pili (so called ebp (endocarditis and biofilm-associated pili)) and cytolysin [3]. Furthermore, its antimicrobial resistance seems to be strongly linked to its capacity to form biofilms [4]. A biofilm is defined as a growth mode of bacteria, bonded tenaciously to a substrate or to an interface or to each other, immersed in a self-produced extracellular polymeric substance (EPS). It has been pointed out that bacteria living in biofilms are phenotypically different from planktonic ones, at least in growth rates and gene transcription [5]. Theoretically, EPS offers protection against various environmental stresses, such as alkaline pH, dryness, high concentrations of salts or lack of nutrients for long periods. Indeed, the bacterial removal from a biofilm is approximately 1000 times more difficult than in planktonic state [6]. The success of endodontic therapy lies, therefore, in the ability to eradicate bacterial biofilms. The complex and unpredictable nature of the anatomy of the root canal system, comprised of accessory canals, isthmi, side canals, and apical deltas, makes the complete removal of bacterial biofilms difficult. Therefore, adequate irrigation is crucial to disinfecting those areas that may not be cleaned sufficiently by instruments.

Conventional syringe irrigation (SI) is widely accepted. Yet it has been argued that in SI the irrigant may not reach the apical region of the canal [7] nor the dentinal tubules, subsequently allowing the persistence of biofilm and the survival of a significant number of viable bacteria, even when the apical preparation is considered to be “complete” [8].

Recently, laser-activated irrigation (LAI) has been proposed as an alternative method to achieve cleaning and disinfection of the root canal system being much more efficient than SI or passive ultrasonic irrigation (PUI) [9]. The LAI mechanism of action consists in the generation of cavitation bubbles through the high absorption of the laser energy by water. This is particularly relevant when using Erbium family lasers (Er:YAG: 2980 nm–Er,Cr:YSGG: 2780 nm) [10,11]. A turbulent flow and the subsequent formation of vapor bubbles in the liquid immediately after the Er,Cr:YSGG (2780 nm) laser activation has been demonstrated by Blanken et al. [12]. Bubbles expand during pulse and then implode generating pressure waves that first displace at supersonic speed (shock waves) and later at sonic speed (acoustic waves). This creates shearing forces along the root canal [13]. This offers a significant advantage over conventional SI, where significant effect take place only in the vicinity of the needle [7]. It has been demonstrated that LAI has bactericidal effect [14], improving the elimination of the dentin smear layer [15], and contributing to the elimination of residue from the apical third of the root [13].

Sodium hypochlorite (NaOCl) is the most widely used endodontic irrigant; it has a broad antibacterial spectrum and dissolves dental pulp tissue [16]. It is used at concentrations ranging between 0.5% and 6% to varying degrees of effectiveness. It has been reported that cell damage is directly proportional to NaOCl concentration [17]. Moreover, prolonged contact causes damages to dentin and periodontal ligament cells, involving acute inflammatory reaction and pain [18]. In a previous work, we reported that Er,Cr:YSGG LAI of 0.5% NaOCl increased the bactericidal effectiveness, on planktonic bacteria and young biofilms, in vitro, reaching the same level of antibacterial effectiveness as 5% NaOCl [19]. This should make feasible the use of NaOCl at lower, and subsequently safer, concentrations. The study was conducted in a laboratory condition by using 24-h-old biofilms. Here we evaluate the antibacterial effectiveness of 0.5% NaOCl activated by the Er,Cr:YSGG laser against a 10-day-old *E. faecalis* biofilm ex vivo, in extracted teeth. Effectiveness was estimated by both bacteriological and microscopy approaches

## 2. Results

### 2.1. Bacterial Elimination

**Figure 1:**
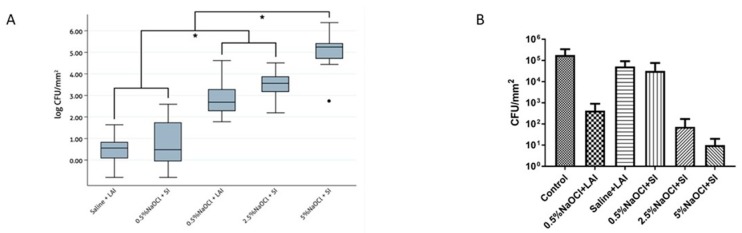
Bacterial counts values of surface area (mm^2^) of *E. faecalis* biofilm after irrigation protocols. Data expressed as median and range. Control means untreated intracanal biofilm. (**A**): Box plot of bacterial reduction. (**B**): bacterial counts after resuspension of surviving microorganisms. * statistically significant difference (P < 0.05)

Bacterial counts and interquartile range (IQR) values are shown in Figure 1. The Shapiro–Wilk test showed that the distribution was not normal (*p* < 0.05) and the non-parametric Kruskal–Wallis test confirmed significant differences between different groups (*p* < 0.05). The bactericidal index is shown in Table 1 and Figure 1. In groups treated with 0.5% NaOCl + LAI and 2.5% NaOCl + SI there was a significant reduction in the number of CFU/mm^2^ (*p* < 0.001). Moreover, reduction of CFU was significantly greater for 5% NaOCl + SI group (*p* < 0.001). Lower efficiencies were achieved by saline solution +‘LAI and 0.5% NaOCl delivered by SI. Values were compared with the ones obtained in inoculated untreated teeth.

### 2.2. Scanning Electron Microscopy (SEM)

**Figure 2:**
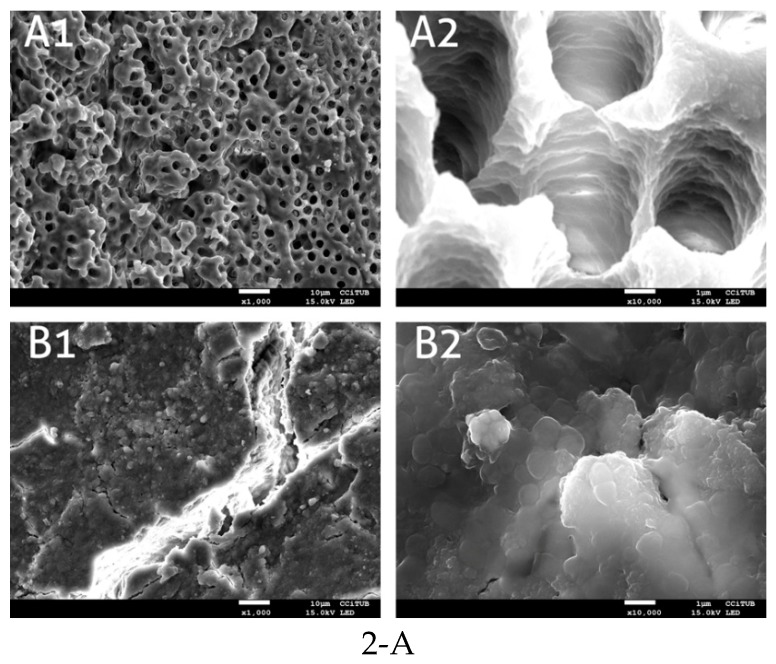

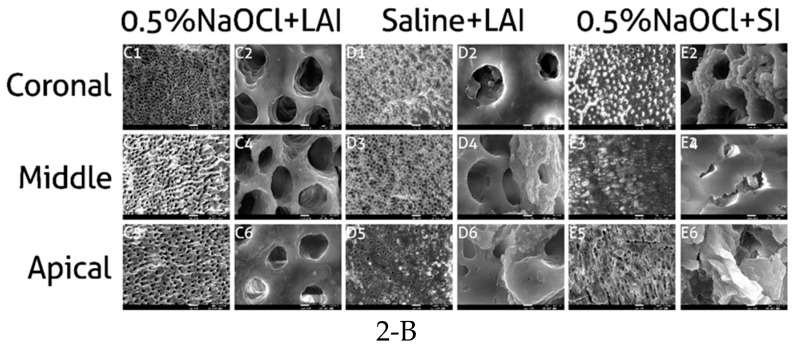
(**A**) SEM images of negative control group (non-inoculated root canals) (**A1**,**A2**) and positive control group (untreated 10 days old biofilm) (**B1**,**B2**). Magnification 1. ×1000; 2. ×10,000. (**B**) SEM images of the coronal, middle and apical thirds of the root canal after different treatments. (**C1**–**C6**): Er,Cr:YSGG laser and 0.5% NaOCl group. (**D1**–**D6**): saline + laser. (**E1**–**E6**): 0.5% NaOCl + SI. Magnification 1, 3, 5: ×1000; 2, 4, 6: ×10,000.

Neither smear layers nor microorganisms were observed on the root canal walls in the negative control; the entrance to the dentin tubules appears open (Figure 2(A1,A2)). After bacterial incubation for 10 days, a heavy and dense biofilm of *E. faecalis* formed on the dentin surface, occluding the dentin tubules (Figure 2(B1,B2)) was seen. The specimens treated with the Er,Cr:YSGG laser and 0.5% NaOCl showed an effective removal of both smear layer and biofilm. The root canal wall displayed open tubules and a clean surface (Figure 2(C1–C6)). In the saline + laser group (Figure 2(D1–D6)) and 0.5% NaOCl + SI group (Figure 2(E1–E6)), the *E. faecalis* biofilm and smear layer were observed on the surface of the root canal walls and inside the dentin tubules, showing that a complete biofilm removal was not achieved. None of the SEM micrographs showed signs of melting.

### 2.3. Confocal Laser Scaning Microscopy (CLSM)

**Figure 3:**
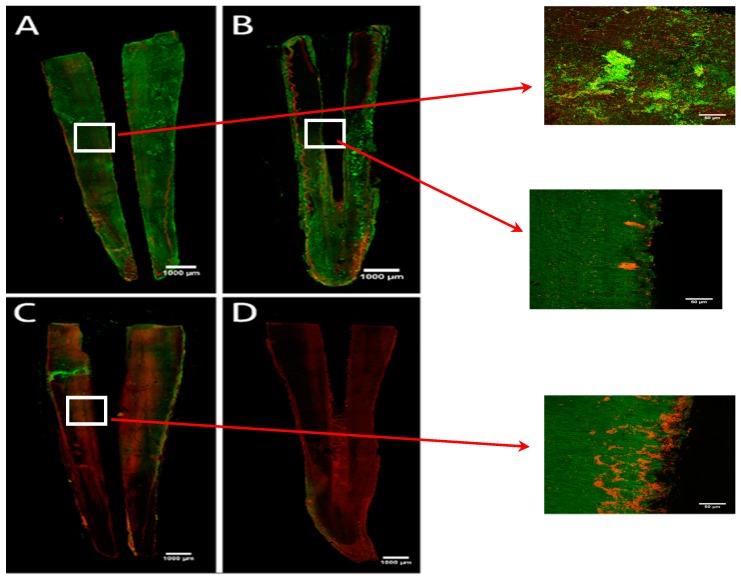
Representative CLMS images of *E. faecalis* biofilm on the surface of the root canal: (**A**) untreated biofilms, (**B**) Saline + Er,Cr:YSGG, LAI (**C**) 0.5% NaOCl + LAI. (**D**) 5% NaOCl +SI. Green: viable bacteria; Red: dead bacteria. Scale bar: 10 μm.

In the control group (Figure 3A) and saline + LAI group (Figure 3B), the CLSM images showed a dense biofilm of *E. faecalis* formed on the dentin surface, formed predominantly by alive bacteria (green). The images revealed the presence of both alive and dead bacteria in the passive irrigation group with 0.5% NaOCl (Figure 3C) with living cells predominating. 

Passive irrigation was not able to reach deep tooth areas. Finally, high bacterial mortality was seen after treatment with the 0.5% NaOCl + LAI group (Figure 3C) which was in agreement with the bacterial count results (Figure 1).

## 3. Discussion

The age of the biofilms used in experimental biology is frequently a matter of discussion. Most research is conducted with 24 or 48-h-old biofilms, while in clinics, it is highly likely that we have to fight much older biofilms (up to 10 days or more). Longer bacterial incubations afford more relevant characteristics thanks to the formation of mature biofilms. The time needed for colonization by *E. faecalis* and biofilm formation varies among the different studies; while some use 24 h of incubation [20], others use 48 h [21], or even much longer incubation periods. We used a 10-day biofilm [22,23] mimicking natural conditions. The bacterial colonization pattern on the dentin and inside the dentin tubules was verified by scanning electron microscopy.

There is no consensus regarding the actual time needed to completely eradicate *E. faecalis* biofilms. Radcliffe et al. [24] demonstrated that 0.5% and 1% NaOCl concentrations need at least 20–30 min to fully remove *E. faecalis* planktonic cells, while 5.25% NaOCl required only 2 min to achieve complete disinfection. In our case, 5% NaOCl released with the SI protocol was significantly more effective at removing *E. faecalis* biofilm than the other treatments with or without activation (*p* < 0.001). The main negative fact is that NaOCl at such high concentrations is extremely irritating to the periapical tissue [18]. Thus, the need to find new alternatives that can make the most of the antimicrobial activity of NaOCl but at less toxic concentrations is a matter of great interest. Other strategies have been used for the same purposes, among them the use of predatory bacteria [25]. The different irrigation techniques have also been extensively studied [26].

It has been reported that laser-activated irrigation significantly enhances the effectiveness of root canal disinfection [14,27]. The expansive shockwaves contribute to the overall photomechanical effect by facilitating access of the irrigant to the apical third of the canals and the deepest areas of the dentin [9]. In addition, the increased movement of NaOCl inside the root canal system increases the contact between the active chlorine molecules and the organic matter and, therefore, improves the chemical effectiveness of the irrigant [28]. However, little is known of the antibacterial effectiveness of low concentrations of NaOCl because most LAI studies have focused on working with high concentrations of NaOCl [14,27,29]. Working on human tooth root canals, we have demonstrated that 0.5% NaOCl combined with the Er,Cr:YSGG laser can effectively disinfect them, being the use of a less toxic concentration of NaOCl feasible. The canals irrigated with LAI 0.5% NaOCl showed a reduction of 3 logarithms in the CFU/mm^2^ count. The SEM revealed a large part of the canal wall and tubules as being free of microorganisms. This finding is encouraging because its effect equaled that of 2.5% NaOCl administered by conventional irrigation. This is relevant as it demonstrates the existence of a synergic effect between the laser and low concentrations of NaOCl. Similar results were obtained by Jaramillo et al. [30], who concluded that the activation of 0.5% sodium hypochlorite with an Er:YAG laser significantly increased its antimicrobial effectiveness. By contrast, Christo et al. [31] observed that, working on a biofilm model with extracted teeth, LAI had a limited potential to increase the antibacterial effect of 0.5% NaOCl. This may be due to the fact that the work was done using an low power Er,Cr:YSGG laser (0.5 W).

Teeth treated with conventional irrigation and 0.5% NaOCl showed a minimum alteration of the *E. faecalis* biofilm since most of dentin tubules exhibited a high number of bacteria. The inability to achieve good results by NaOCl at low concentration without activation stands out the relevant interest of laser energy in the disinfection of the root canal at these low concentrations.

Despite obviously saline is not bactericidal, some bactericidal effects were observed when used as an irrigant with LAI. The related bacterial death may be due to the intense flow action created within the irrigant [31]. Although the combination of activation laser and saline produced an alteration of biofilm, *E. faecalis* remained within the dentin tubules and on the dentin surface. It should be noted that the combination of the laser and saline improved the elimination of the smear layer, supporting the observations made by Di Vito et al. [15].

It has been reported that NaOCl extrusion increases during activation by the laser. Peeters & De Moore [32] demonstrated that the likelihood of extrusion is greater the closer the apex is placed to the optical fiber. Here we performed activation with the optical fiber in the coronal portion of the tooth for the duration of the activation, thereby decreasing the likelihood of irrigant extrusion. Recently, Peeters et al. [33] studied the degree of extrusion of radiopaque contrast medium in 20 teeth with open apex using Er,Cr:YSGG LAI (1 W, 35 Hz). The results showed a total absence of contrast medium in every case, demonstrating the safety of the technique. Nevertheless, extreme caution should be exercised particularly in the vicinity of apical constriction to prevent extrusion. 

It should be taken into account that laser has some disadvantages as is the price of equipment and may have some risks when non-expert dentists use inappropriate parameters. It has been seen that the increase in temperature caused by laser energy can produce undesirable effects in the dentin, such as cracks, small fissures or carbonization [34]. In our experiments, the laser fiber was used away from the dental apex and never made contact with the canal walls, thus protecting the structure from possible thermal damage. This was confirmed in the SEM analysis, where the undamaged dentin can be observed following treatment. In a 10-day-old *E. faecalis* biofilm in extracted teeth, the Er,Cr:YSGG LAI proved to be able to improve the antibacterial properties of 0.5% NaOCl after 60 s of activation, reaching the same level of effectiveness as 2.5% NaOCl. This is of great clinical interest, because it demonstrates that a lower concentration of NaOCl may be useful diminishing undesired secondary effects. 

## 4. Materials and Methods 

### 4.1. Specimens 

The study protocol was approved by the Clinical Research and Ethics Committee of the University of Barcelona (#2016-23). A total ninety-one human single-rooted teeth extracted for therapeutics purposes were collected. To eliminate periodontal ligament remnants and calculus from the root surface, the specimens were subjected to cleaning using endodontic tips (ProUltra Zirconium Nitride, Dentsply Maillefer, Ballaigues, Switzerland) and a Gracey 7/8 curette (Hu-Friedy, Chicago, IL, USA). The specimens were stored in formalin solution 10% at 4 °C until use.

All teeth were decoronated under the cemento-enamel junction to a standardized length of 14 mm as described by Christo et al. [31]. A coronal reservoir of 5 mm was created with a #016 cylindrical diamond bur (Komet, Rock Hill, SC) at the entrance of the root canal. Apical permeability and single canal confirmation were checked with a K-File #10 (Dentsply Maillefer, Ballaigues, Switzerland). The working length (WL) was determined by reducing 1 mm from the point at which the K-File #10 was visible through the apical foramen. The canals were instrumented using the conventional sequence of 0.02 taper files up to the master K-File #45 (Dentsply Maillefer^®^, Ballaigues, Switzerland). After the use of each instrument, the root canals were irrigated with 1 mL of 2.5% NaOCl using a syringe and a 30-gauge side-vented needle (Becton Dickinson, Madrid, Spain) to the WL. The canals were irrigated with 1 mL ethylenediaminetetraacetic acid (EDTA) (Denta Flux, Madrid, Spain) for 1 min, followed by 1 mL of 2.5% NaOCl and 1 mL of saline. The apical foramen and the root surface were sealed with a double layer of nail polish (02 Nail Polish, Depend Cosmetic AB, Halmstad, Sweden) to prevent the extrusion of the irrigant through the apex and to provide a closed system [31]. The dental roots were stored in Eppendorf tubes and autoclaved at 121 °C for 17 min.

### 4.2. Enterococcus Faecalis Biofilm Formation

*E. faecalis* ATCC 29212 (American Type Culture Collection) was maintained by weekly subculturing on trypticase soy agar (TSA) plates (Scharlau, Sentmenat Barcelona, Spain). A single colony was inoculated in 40 mL of tryptic soy broth (TSB) medium and incubated at 37 °C. After 24 h of incubation, the culture was diluted 100 times in fresh TSB, and adjusted spectrophotometrically (Unicam UV-2 at 600 nm) at OD600 = 1.3 (i.e., 7.8 × 108 colony-forming units CFU/mL). Root surfaces were coated with 0.01% (*w*/*v*) poly-L-lysine hydrobromide (Sigma-Aldrich, Dorset, UK) to enhance bacterial adhesion and inoculated with 10 µl of bacterial culture using a 30-gauge syringe and needle (Becton Dickinson, Madrid, Spain). The dental roots were placed in Eppendorf tubes and incubated at 37 °C for 10 days. Re-inoculation at days 1, 4 and 7 were performed to ensure the presence of live bacteria during the incubation period [20]. Finally, the inner part of the root canal was gently washed with 1 mL of Ringer’s ¼ solution to remove the free-floating microbes and liquids. 

### 4.3. Experimental Procedures

The teeth were randomly distributed into seven groups (*n* = 13). Each group was submitted to a different treatment: (I) 0.5% NaOCl + Er,Cr:YSGG LAI (II) Saline + Er,Cr:YSGG LAI (III) 0.5% NaOCl + SI (IV) 2.5% NaOCl + SI (V) 5% NaOCl + SI (VI) Positive control (no treatment) (VII) Negative control (no bacteria). Eighteen teeth were then randomly divided in to two subgroups for investigation with CLSM (*n*= 8) and SEM microscopy (*n* = 10) techniques.

The SI protocol was done by slowly placing up to 5 mL of the irrigant into the WL and allowing it to act for 60 s. Finally, canals were irrigated with 2 mL of sodium thiosulfate 5% to inactivate the remaining NaOCl and washed with 1 mL of saline.

Laser irradiation took place using an Er,Cr:YSGG pulsed laser (Waterlase iPlus; Biolase Technology, Irvine, CA, USA) at a wavelength of 2780 nm. The laser operating parameters were 1 W of power, 10 Hz of repetition frequency, 100 mJ energy per pulse and 140-μs of pulse duration. The coaxial water spray from the Gold Handpiece (Biolase Technology, Irvine, CA, USA) was switched off throughout the treatment. An RFT 2 tip (200 μm in diameter, 21 mm long, calibration factor >0.55, Endolase, Biolase Technology, Inc. Irvine, CA) was used. It is a conical tip with a 50° angle, designed for endodontic treatment. The real power was 0.55 W at 10 Hz, 55 mJ per pulse. Autoclaved tips were positioned only in the coronal reservoir during activation. During the laser irradiation cycles, irrigant was added as the coronal reservoir was empty; thus, LAI was permanently carried out in the presence of irrigant. The Er,Cr:YSGG laser was activated for 30 s, followed by a rest phase of 30 s and ending with 30 s of activation (60 s of activation in total). Finally, sodium thiosulfate and saline were used as before. 

### 4.4. Bacterial Count

Bacteria were suspended in Ringer ¼ by using an ultrasonic cleaner (Raypa, Barcelona, Spain) at maximum power followed by vortex agitation for 3 min. Colony-forming units (CFU) per ml were enumerated by plating tenfold serial dilutions on TSA plates incubated for 24 h at 37 °C. Values were transformed to CFU/mm^2^.

### 4.5. Scanning Electron Microscopy (SEM)

A water-cooled diamond cutting blade mounted on a precision cutting machine (Mecatome, Persi, France) was used to cut the specimens longitudinally. The two parts were mounted on the microscope supports by means of a conductive double-sided adhesive disc. Next, they were covered with a fine graphite layer to improve their electrical conductivity (Emitech K950X high vacuum evaporator) and examined in a Jeol J-7100F scanning electron microscope (Tokyo, Japan) at 15.0 kV. Visualizations were done at 1000× and 10,000× to assess the bacterial biofilm and the smear layer in the coronal (10–12 mm from the apex), middle (6–7 mm from the apex) and apical (1–2 mm from the apex) parts. 

### 4.6. CLSM

To stain the biofilms, a mixture of SYTO 9 and propidium iodide prepared at a dilution ratio of 1:2 (1.5 µL of SYTO 9 and 3 µL of propidium iodide (PI) in 1 mL of Ringer ¼) was applied to the whole biofilm. After 30 min of incubation in the dark at 37 °C, the stained biofilms were washed once with Ringer ¼ to remove nonspecific staining. Fluorescence was observed using a Zeiss LSM 880 spectral confocal laser scanning microscope (Carl Zeiss, Jena, Germany) equipped with a 488-nm argon laser and 561-nm diode lasers. The reconstruction of whole teeth was performed with stitched images of different focal planes obtained with 10x magnification objective (0.45 numerical aperture) using the Zen black software (Carl Zeiss, Jena, Germany). The zoom images were obtained with 40× immersion oil objective (1.3 numerical aperture). The image resolution was 1024 × 1024 pixels with both magnifications. ImageJ software (National Institutes of health, Bethesda, MD, USA) and IMARIS software (Bitplane AG, Zurich, Switzerland) were used to obtain LSM images. 

### 4.7. Statistical Analysis

Statistical analysis was performed with Stata14 (StataCorp^®^, College Station, TX, USA). Data were transformed logarithmically. The bactericidal effects were expressed as a bactericidal index (BI); i.e., the difference between the logarithm of the bacterial counts of the control and the treatment groups. The normality of the scale variables was explored using the Shapiro–Wilk test and the visual analysis of the P-P graph and the box plot. When normality was rejected, both the interquartile range (IQR) and the median were calculated. A statistical analysis was performed to compare the UFC/mm^2^ values using the Kruskal–Wallis nonparametric test and Bonferroni’s post hoc test for multiple comparisons. The level of significance was set at *p* < 0.05.

## Figures and Tables

**Table 1:** Multiple independent variables on the bactericidal index. Statistically significant differences were set at *p* < 0.05. LAI, laser-activated irrigation; SI, syringe irrigation.

Group	Treatment	0.5% NaOCl + LAI	Saline + LAI	0.5% NaOCl + SI	2.5% NaOCl + SI
1	0.5% NaOCl + LAI				
2	Saline + LAI	<0.001			
3	0.5% NaOCl + SI	<0.001	0.999		
4	2.5% NaOCl + SI	0.3167	<0.001	<0.001	
5	5.0% NaOCl + SI	<0.001	<0.001	<0.001	<0.001
